# New Times, New Ways: Exploring the Self-Regulation of Sport during the COVID-19 Pandemic and Its Relationship with Nostalgia and Well-Being

**DOI:** 10.3390/bs13030261

**Published:** 2023-03-15

**Authors:** Heetae Cho, Mun Yip Kinnard Chen, Hyoung-Kil Kang, Weisheng Chiu

**Affiliations:** 1 Department of Sport Science, Sungkyunkwan University, Suwon 16419, Republic of Korea; 2 Department of Physical Education and Sports Science, Nanyang Technological University, Singapore 639798, Singapore; 3 Department of Psychology, Nanyang Technological University, Singapore 639798, Singapore; 4 Department of Physical Education, Kyungnam University, Changwon 51767, Republic of Korea; 5 Lee Shau Kee School of Business and Administration, Hong Kong Metropolitan University, Hong Kong, China

**Keywords:** nostalgia, sport activity, COVID-19, self-regulation, subjective well-being

## Abstract

Coronavirus disease (COVID-19) has negatively affected individuals’ participation in sport activities, while sport participation is an important regulator of well-being. The current study investigated the effects of the nostalgia for sport activities and self-regulation of sport activities on subjective well-being. A total of 302 responses were collected from participants who had engaged in sport activities before the lockdown period. The data were analyzed using partial least squares structural equation modeling (PLS-SEM). The findings showed that nostalgia positively affected the self-regulation of sport and subjective well-being. In addition, self-regulation of sport was positively associated with subjective well-being. Based on the findings of this study, policymakers can implement interventions that promote an individual’s feelings of nostalgia, as it might lead them to engage in sport or promote self-regulation.

## 1. Introduction

Coronavirus disease (COVID-19) is a highly infectious virus that caused a global pandemic [1]. By 30 June 2021, this respiratory virus had caused more than 3.9 million deaths worldwide [2]. Many countries had adopted public health measures, such as social distancing, hygiene practices, and even complete lockdowns for the general population to reduce the spread of this virus and the strain on healthcare systems [3]. For example, the Singapore Parliament passed the COVID (Temporary Measures) Act on 7 April 2020, and implemented a similar set of public health measures for “preventing, protecting against, delaying, or otherwise controlling the incidence or transmission of COVID-19 in Singapore” [2] (p. 1). Singapore was in Phase Two during the conceptualization of this study in November 2020 [4]. During this phase, several activities resumed, and more facilities could remain open, although certain health protection measures remained in effect [4]. However, owing to various health measures and restrictions on sport and sport facilities, engaging in sport activities was a challenge. In particular, under the “Advisory for the Resumption of Sports and Physical Exercise and Activity for Phase Two”, individuals were required to maintain a physical distance of two meters between each other while exercising or playing sports [5]. Sport events that required close contact with others were either canceled or modified to accommodate the health measures. In other words, the imposition of several restrictive health measures made it challenging to engage in sport activities in Singapore during Phase Two. Thus, the increased difficulty in engaging in sport might have reduced individuals’ level of sport participation [6].

These increased difficulties in engaging in sport activities might cause some people to feel nostalgic toward the past when they were able to participate in sport without any restrictions but are now unable to do so. Batcho [7] showed that feelings of nostalgia arise during times of transition as a way of finding continuity. Similarly, Gibbs and Egermann [8] found that nostalgia increases during transitional periods such as the COVID-19 lockdown and serves as a form of psychological resource that provides a buffer against the anxieties and difficulties that accompany such changes. It is essential to study nostalgia, as it has been associated with numerous psychological responses [9]. For instance, individuals who reported a greater intensity and frequency of nostalgia tended to report greater meaning in life [10] and positive emotionality [11]. In addition, researchers found that feelings of nostalgia increase an individual’s optimism, self-esteem, social-connectedness, and meaning in life [10,12,13].

Although previous studies investigated how nostalgia influences various psychological outcomes [7,8,9,10,11,12], there have been only a few studies on the effect of nostalgia on sport participants’ self-regulation and well-being by considering the novelty of the COVID-19 pandemic and the consequent restrictions placed on sport activities [14]. Therefore, this study aimed to examine the relationships among an individual’s nostalgia for sport activities, self-regulation of sport activities, and subjective well-being during the COVID-19 situation. This study contributes to enhancing our understanding of the role of nostalgia and its effects on individuals’ self-regulation of sport behavior and well-being. In addition, the findings of this study can help policymakers adjust and adapt the restrictions to optimize and improve individuals’ well-being.

## 2. Literature Review

### 2.1. Broaden-and-Build Theory of Positive Emotions

Fredrickson [15] asserted that experiences of positive emotions broaden individuals’ thought–action repertoires, which builds their enduring personal resources over time. Two central aspects of this theory are: (1) the broadening of thought–action repertoires and (2) the building of one’s personal resources. The first proposition asserts that positive emotions broaden an individual’s awareness and encourage new, exploratory thoughts and behaviors. Previous literature has shown that individuals experiencing positive emotions often exhibit thought patterns that are creative [16], flexible [17], and open to new information [18]. Such individuals often have a “broad, flexible cognitive organization and the ability to integrate diverse material” [19] (p. 89). Kahn and Isen [20] found that individuals who experience positive emotions also have an increased preference for novelty and are more open to trying new behaviors. This leads to the second proposition, which asserts that the broadening of an individual’s thought–action repertoires builds their personal resources.

According to the existing literature, when an individual’s attention and cognition are broadened, it permits flexible, creative, and integrative thinking, which can enhance their coping resources [21]. For instance, individuals who experience positive emotions during bereavement are more likely to develop long-term plans and goals, both of which predict greater psychological health [22]. Fredrickson and Joiner [23] showed that individuals who experience more positive emotions tend to be more resilient during adversity due to their broad-minded coping. These improved coping resources then increase the likelihood of experiencing positive emotions, which further improves their personal resources, possibly leading to an upward spiral of improved well-being and coping resources [15,23]. That is, positive emotions contribute to personal well-being [15]. Nostalgia is considered a bittersweet emotion but is still predominantly positive [11]. Thus, nostalgia, as a positive emotion, might broaden an individual’s thought–action repertoires and help build personal resources.

### 2.2. Nostalgia for Sport Activities

The increased difficulty in engaging in sport activities might have led some individuals to experience nostalgia for the pre-COVID-19 state of sport, where access to sport activities was not limited by any mandatory restrictions. Cho et al. [24,25] defined nostalgia as a sentimental longing for a positive past; comparisons with negative current or future situations can induce feelings of nostalgia. According to Cho et al.’s [24] classification of nostalgia, nostalgia consists of two dimensions: (1) the purpose of nostalgia and (2) the structure of nostalgia. The ‘purpose of nostalgia’ dimension encompasses an individual’s priorities and values that are based on past experiences. The ‘structure of nostalgia’ dimension asserts that nostalgia can be evoked by objects or interpersonal relationships. Object-based nostalgia includes places, symbols, or facilities, whereas interpersonal-relationship-based nostalgia involves social experiences with other people [24]. 

This classification is further divided into four factors: (1) experience, (2) socialization, (3) personal identity, and (4) group identity. The first factor, experience, refers to the fact that nostalgic feelings can be induced by one’s past experiences [24]. Reminiscing about certain sport athletes, teams, facilities, or atmospheres in the past can evoke a sense of nostalgia [26]. The second factor, socialization, states that nostalgia can be experienced through past experiences of social interactions with others [27]. For instance, recollecting past sport experiences with friends and family members can evoke feelings of nostalgia. The third factor, personal identity, states that an individual’s identification with sport can generate nostalgia. In other words, individuals who view themselves as sport fans can have nostalgic feelings regarding their roles and identities as fans [28]. Last, similar to the previous factor, the fourth factor, group identity, states that individuals with group identities in sport, such as being in a sport fan club, can experience nostalgic feelings when recollecting information about the past [29]. More specifically, it asserts that nostalgic feelings are evoked by the norms, rituals, and culture of the social group. 

The current study selected and adapted this classification of nostalgia since it focused on nostalgia in the context of sport [24]. As previously mentioned, the increased difficulty in engaging in sport activities might have led individuals to experience nostalgia about the pre-COVID-19 state of sport activities [14]. In other words, protective health measures might have led them to long for the past when no restrictions were imposed on sport activities.

### 2.3. Self-Regulation of Sport Activities and Subjective Well-Being

The broadening and building of personal resources can be measured by an individual’s self-regulation of sport activities. Self-regulation refers to the process by which individuals engage in goal-directed behavior through strategies such as behavioral monitoring, selective processing of information, and self-evaluation [30,31]. That is, the self-regulation of sport activities indicates a personal resource that an individual may have developed and involves strategies that an individual can learn to employ to effectively manage goal-directed behavior [31]. These strategies can be considered personal resources that enable individuals to effectively reach their goals. Therefore, the self-regulation of sport activities can serve a broadening function under the broaden-and-build theory of positive emotions [14], as it encourages an individual to determine new self-regulatory sport activities.

Based on the broaden-and-build theory [14], feelings of nostalgia, as a predominantly positive emotion, might lead individuals to self-regulate their sport activity by identifying new sport activities (broadening function) and building new sources of sport activities (building function). Previous studies have shown that feelings of nostalgia motivate future-oriented behaviors [32,33], such as engagement in sport activity and healthy eating [34,35]. Kersten et al. [34] explained that nostalgia shifts an individual’s mindset toward improving future outcomes, of which their health is one important domain. Thus, individuals who experience stronger feelings of nostalgia often have higher levels of health optimism, develop stronger health-related attitudes, and consequently adopt better health habits such as engaging in sport activity [33,34]. Additionally, Bocincova et al. [36] found that feelings of nostalgia lead an individual to be in a psychological state of pursuit rather than avoidance. In other words, individuals experiencing feelings of nostalgia are more likely to approach and face any potential problems instead of avoiding them. This indicates that when faced with restrictions on sport activities, individuals experiencing feelings of nostalgia are more likely to engage in and identify new self-regulatory sport behaviors instead of avoiding the problem of sport restrictions. Therefore, nostalgia might increase an individual’s self-regulation of sport activities. Thus, this study hypothesized the following:

**H1:**  *Nostalgia for sport activities is positively related to the self-regulation of sport activities.*

Based on the broaden-and-build theory of positive emotions, building personal resources, such as the self-regulation of sport activities, can result in improved subjective well-being. Subjective well-being (SWB) refers to individuals’ appraisals and evaluations of their lives [37]. It includes cognitive judgments (e.g., life satisfaction) and emotional aspects (e.g., positive or negative emotions) [37]. Additionally, the personal well-being index—adult (PWI-A) can be used to measure SWB [38]. It is essential to study SWB because higher levels of SWB have been associated with numerous desirable outcomes such as higher levels of happiness [39], improved work performance [40], and enhanced physical health and longevity [41].

According to the broaden-and-build theory [15], positive emotions can improve an individual’s subjective well-being through the development of personal resources. As mentioned previously, nostalgia is a future-oriented emotion [33] that can improve well-being through the adoption of health-related attitudes. Furthermore, it can affect new health-related behaviors, such as sport activity [34]. Considering the broaden-and-build theory [14], engagement in sport activity is a personal resource that can improve an individual’s well-being. Moreover, nostalgia enhances an individual’s subjective well-being [42] by building other personal resources [10,43] that can serve as a psychological buffer against adversity [44]. This is consistent with existing literature that has shown the association of nostalgia with numerous psychological benefits [9], such as positive emotionality [45], optimism, and self-esteem [12,13]. Therefore, this study proposed the following hypothesis:

**H2:**  *Nostalgia for sport activities is positively related to subjective well-being.*

Fredrickson [15] noted that personal resources can lead to improved well-being. For instance, self-regulation positively predicts long-term health [46] and educational outcomes, including academic achievement among adolescents [47]. Moreover, it is inversely related to poor health outcomes such as obesity [48]. This suggests that the self-regulation of sport activities can be a personal resource that has been built up, and improvement in self-regulation can promote physical and psychological health [49]. Previous studies have shown that individuals who can self-regulate and regularly engage in sport activity are often healthier and have higher levels of subjective well-being [50,51,52]. In a recent meta-analysis of sport activity and subjective well-being, Buecker et al. [53] found that engaging in sport activity improves an individual’s subjective well-being regardless of their prior fitness level and the type of sport activity they engaged in. Furthermore, other studies have shown that this positive relationship between regular sport activity and subjective well-being persists among individuals in different age groups [54,55]. Considering the existing literature, we proposed the following hypothesis:

**H3:**  *The self-regulation of sport activities is positively related to subjective well-being.*

## 3. Methods

### 3.1. Data Collection

This study collected data from individuals aged 21 and above who participated in sport activities before the COVID-19 lockdown. In this study, we defined sport activity as a physical activity engaged in for pleasure [56]. Data were collected in Singapore using a snowball sampling method from 1 February to 31 May 2021. Specifically, online survey forms were disseminated through multiple social media platforms (e.g., Facebook and WhatsApp) using Google Forms. In addition, participants were encouraged to share the survey link with their friends and family members who were eligible for this study. Participants of the online survey have to be at least 21 years old in the year 2021. Participants were informed about the voluntary nature of participation and that they could withdraw from the study at any time. Furthermore, they were informed that there would be no monetary benefit for their participation. Participants spent approximately 15 min on average completing the questionnaire. The response rate was estimated to be approximately 20%; finally, this study recruited 302 participants from Singapore.

### 3.2. Measures

The survey was conducted using Google Forms. The questionnaire consisted of six sections that collected (1) participants’ demographic information, while also assessing their levels of (2) nostalgia for sport activities, (3) self-regulation for sport activities, and (4) subjective well-being. The questionnaire included items from existing scales that measured these constructs with minimal modifications to suit the context of sport activities. 

#### 3.2.1. Nostalgia for Sport Activities

Cho et al.’s [57] leisure nostalgia scale was borrowed and modified to measure nostalgia for sport activities instead of leisure activities. For instance, the term “leisure” was replaced with “sport activity” in this study. This 33-item scale measures five subfactors: (1) Nostalgia as Sport Experience (e.g., Remembering a sport activity that I enjoyed evokes my nostalgic feelings), (2) Nostalgia as Environment (e.g., The architectural design of my favorite sport activity place evokes my nostalgic feelings), (3) Nostalgia as Socialization (e.g., Positive feelings for building friendships with others during my favorite sport activity evokes my nostalgic feelings), (4) Nostalgia as Personal Identity (e.g., Pride in being a fan of my favorite sport activity evokes my nostalgic feelings), and (5) Nostalgia as Group Identity (e.g., Pride of being a part of my group at the sport activity place evokes my nostalgic feelings). The responses on this scale were scored using a 7-point Likert scale ranging from 1 (Strongly Disagree) to 7 (Strongly Agree). 

#### 3.2.2. Self-Regulation of Sport Activities

Fleury’s [58] index of self-regulation was borrowed to measure an individual’s self-regulation of physical activity. This 9-item scale measures three factors: (1) Reconditioning (e.g., I think of the benefits of regular sport activity), (2) Stimulus Control (e.g., I keep track of the ways that I can stay active in sport), and (3) Behavioral Monitoring (e.g., I have learned new habits that help me participate in sport activity). Participants were required to indicate their level of agreement with the items using a 6-point Likert scale ranging from 1 (Strongly Disagree) to 6 (Strongly Agree).

#### 3.2.3. Subjective Well-Being

The personal well-being index—adult (PWI-A) developed by the International Wellbeing Group [38] was used to measure participants’ subjective well-being. This scale comprises seven items: (1) Standard of Living Domain (e.g., How satisfied are you with your standard of living?), (2) Personal Health Domain (e.g., How satisfied are you with your health?), (3) Achieving in Life Domain (e.g., How satisfied are you with what you are achieving in life?), (4) Personal Relationship Domain (e.g., How satisfied are you with your personal relationships?), (5) Personal Safety Domain (e.g., How satisfied are you with how safe you feel?), (6) Community-Connectedness Domain (e.g., How satisfied are you with feeling part of your community?), and (7) Future Security Domain (e.g., How satisfied are you with your future security?). The items were scored on an 11-point Likert scale ranging from 0 (No satisfaction at all) to 10 (Completely satisfied).

### 3.3. Data Analysis

Data were analyzed using partial least squares structural equation modeling (PLS-SEM) via SmartPLS 4 [59]. Before the analysis, the data were screened to identify missing values and outliers. Missing values were treated using the expectation-maximization (EM) algorithm. Standardized z-scores were used to identify univariate outliers, whereas the Mahalanobis distance was used to identify multivariate outliers. Subsequently, Anderson and Garbing’s [60] two-step approach was used to investigate the proposed model. First, the measurement was examined to evaluate the scale reliability and validity. Second, the structural model was carried out to test the hypotheses.

## 4. Results

No univariate or multivariate outliers were detected in the data screening, leaving 302 responses for further analysis. Of the 302 respondents, 49.0% (*n* = 148) were women and 51.0% (*n* = 154) were men. The participants’ average age was 24 years (SD = 4.034), ranging from 21 to 56 years. Most participants were Chinese (*n* = 262, 86.8%), followed by Malay (*n* = 16, 5.3%), Indian (*n* = 16, 5.3%), Latina (*n* = 3, 1.0%), Filipino (*n* = 3, 1.0%), and Eurasian (*n* = 16, 0.7%). 

### 4.1. Measurement Model

The measurement model was first assessed to determine the indicator reliability, composite reliability (CR), and internal consistency reliability (Cronbach’s α). However, it was found that one item of ‘nostalgia as environment’ and two items of ‘subjective well-being’ revealed low indicator loadings (<0.60). Therefore, these items were removed to improve the overall reliability and validity of the measures without compromising the meaning of the constructs [61]. As shown in Table 1, reliability was supported due to the adequate internal consistent reliability (α > 0.70) and CR (>0.50). The average variance extracted (AVE) values were examined to evaluate convergent validity. The AVE values ranged from 0.865 for reconditioning to 0.951 for behavioral monitoring, indicating good convergent validity (AVE > 0.50) [62] (Table 1). Moreover, the disjointed two-stage approach [63] was applied to evaluate the second-order constructs of nostalgia and self-regulation. Table 2 showed that all the indicators (i.e., α, CR, and AVE) exceeded the suggested values [61]. Finally, discriminant validity was assessed by the heterotrait–monotrait ratio of correlations (HTMT) to see if the HTMT coefficients were greater than 0.85 [64]. Table 3 indicated the acceptable discriminant validity of the measures in this study. 

### 4.2. Structural Model

**Figure 1:**
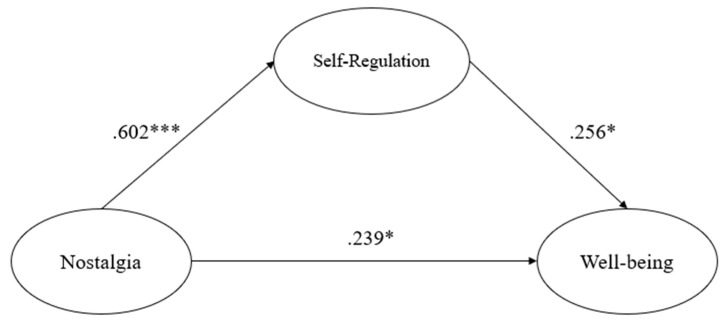
Standardized coefficients of the structural equation model (Note. * *p* < 0.05; *** *p* < 0.001).

The relationships between the participants’ COVID-19 nostalgia for sport activities, self-regulation of sport activities, and subjective well-being were examined (Table 4). The results indicated that nostalgia was positively associated with the self-regulation of sport (H1: *β* = 0.602, SE = 0.083, *t* = 7.258, *p* < 0.001) and subjective well-being (H2: *β* = 0.239, SE = 0.117, *t* = 2.049, *p* < 0.05). Moreover, the self-regulation of sport was positively associated with subjective well-being (H3: *β* = 0.256, SE = 0.105, *t* = 2.424, *p* < 0.05). The predictors explained 44.9% and 24.5% of the variance in the self-regulation of sport activities and subjective well-being, respectively (Figure 1). Additionally, mediation analysis revealed that nostalgia had a significant indirect effect on subjective well-being (*β* = 0.154, SE = 0.063, *t* = 2.456, *p* = 0.014).

## 5. Discussion

This study investigated the relationships between the nostalgia for sport activities, self-regulation of sport activities, and subjective well-being based on the broaden-and-build theory of positive emotions [15]. More specifically, nostalgia for sport activities was found to positively predict an individual’s self-regulation of sport activities (H1). This result is consistent with the broaden-and-build theory of positive emotions, which asserts that individuals who experience positive emotions are more likely to build new and enduring personal resources [15]. These resources include determining new ways to engage in sport activities. Furthermore, FioRito and Routledge [33] noted that nostalgia is a future-oriented emotional experience that motivates certain behaviors, emotions, and goals that enhance an individual’s future outcomes. For instance, nostalgia increases certain motivation-related emotions that promote future-oriented actions such as inspiration [45], optimism [10], and a sense of purpose in life [12]. Additionally, Routledge et al. [65] found that individuals’ nostalgic memories often contain a sense of hope for the future. Kersten et al. [34] found that hopefulness for one’s health induced by feelings of nostalgia is also associated with stronger intentions to engage in sport activity or a healthy diet. In other words, nostalgia triggers emotional states that promote actions and health [33]. Similarly, a recent neurological study by Bocincova et al. [36] found that feelings of nostalgia shift individuals toward approach-related psychological states.

Our results supported the hypothesis that nostalgia would be positively related to subjective well-being (H2). This is consistent with numerous previous studies that showed nostalgia to be significantly and positively associated with an individual’s subjective well-being in many domains [9], such as enhanced meaning in life [10] and positive emotionality [11]. In the context of sport, FioRito and Routledge [33] have asserted that the future-oriented nature of nostalgia indirectly improves an individual’s well-being by promoting future-oriented emotions and behaviors such as caring for their physical health [34]. However, it should also be noted that nostalgia can improve an individual’s subjective well-being through other aspects as well. For instance, Rao et al. [43] stated that nostalgia could improve an individual’s subjective well-being by constructing or strengthening their meaning in life. Meaning in life can serve as an essential psychological coping resource and can thus be both an indicator of [66] and a contributor to an individual’s higher subjective well-being [44]. Routledge et al. [65] noted that nostalgia promotes feelings of purpose and meaning in life, as it involves the perception of important life experiences with certain objects or significant individuals.

Finally, self-regulation positively predicted subjective well-being (H3). This is consistent with the broaden-and-build theory of positive emotions, which states that the development of new personal resources, such as finding new sport activities, can enhance an individual’s well-being [15]. This is also consistent with numerous existing studies that have shown that regular engagement in sport activity promotes an individual’s health and subjective well-being [50,51,52,67,68]. Furthermore, the previous literature has shown that certain types of motivation toward sport activity have a differential impact on an individual’s well-being [69]. More specifically, autonomous motivation toward sport activity has been more strongly associated with higher levels of subjective well-being through numerous domains, such as increased happiness [70], quality of life [71], and physical self-worth [72]. As mentioned previously, Yeom et al. [31] indicated that an individual’s self-regulatory sport behavior involves autonomous motivation. Thus, previous research on the relationship between autonomous motivation toward sport activity and subjective well-being explains the positive relationship between the self-regulation of sport activities and subjective well-being.

### 5.1. Practical Implications

The current study has some practical implications in the context of the self-regulation of sport behaviors during the COVID-19 pandemic. First, policymakers should implement interventions to promote feelings of nostalgia toward the pre-lockdown state of unrestricted access to sport activity as it might lead them to engage in sport activities or promote the self-regulation of sport activities.

Second, the findings suggest that it is essential for an individual to begin participating or continue to participate in sport activity, even during a pandemic, because it can significantly affect their well-being. In other words, one should seek alternative sport activities that do not violate the restrictions. For instance, even though contact sport was not allowed during Phase 2 in Singapore, other sport activities, such as running or exercising in public parks, were allowed [5]. Thus, individuals should identify alternatives to reach and maintain an optimal level of sport activity that meets the international guidelines provided by the World Health Organization [73]. Lastly, policymakers should explore and allow alternative sport activities that are relatively safe for the general population. Additionally, they should encourage the general population to continue their engagement in sport activities that are within the limits of restrictions. These self-regulatory sport behaviors would then enhance the well-being of the general population, an aspect that is crucial during a pandemic situation that negatively impacts individuals’ well-being [1].

### 5.2. Limitations and Future Research

Although the current study has yielded some significant insights into the nature of people’s sport behaviors and well-being, it has a few limitations. First, this study utilized a cross-sectional design that measured individuals’ sport behaviors and subjective well-being at a specific time point, namely, during Phase Two of the restrictions. Thus, changes in individuals’ sport behaviors and subjective well-being cannot be traced across different phases to determine how restrictions on sport activity might have affected the two aforementioned constructs. Therefore, future research should adopt a longitudinal design to trace the changes in individuals’ sport behaviors and subjective well-being across different phases of restrictions. This would provide further evidence and an understanding of how factors such as nostalgia, the self-regulation of sport activities, and subjective well-being interact with one another and change depending on the environmental context. Next, this study did not include items related to respondents’ sport experience (e.g., weekly exercise frequency, duration, and intensity) in the questionnaire. Moreover, most participants were young adults (mean age = 24.00) and Chinese (86.8%). Therefore, the present study cannot be generalized to other cultures and a larger population. Future research should compare the current results to samples from other populations or cultures to determine whether the findings persist across different populations. Cross-cultural studies could also be conducted to provide additional support for the present findings. Finally, the current study utilized a quantitative approach. Thus, future research can employ a qualitative approach to provide a deeper understanding of the factors that could affect individuals’ self-regulation of sport activities during pandemics [74]. It would further provide a more contextual understanding of the impact of different restrictions imposed on sport activities, particularly on individuals’ self-regulatory sport behaviors.

## 6. Conclusions

In summary, this study explored the relationships between the nostalgia for sport activities, self-regulation of sport activities, and subjective well-being in the context of sport restrictions during Phase 2 in Singapore. Our results supported all three hypotheses. This suggests that policymakers should investigate the restrictions on sport activities, encourage the general population to continue to maintain or increase their sport activity level, and provide alternative sport activities. Unfortunately, the proposed findings are deemed to be less plausible than is reasonably accepted, given the poor model fit. Therefore, future studies should improve the data by improving the data collection procedures or proposing other models for a better data fit.

## Figures and Tables

**Table 1:** Assessment of measurement model: first-order constructs.

First-Order Construct	Items	Factor Loadings	Cronbach’s α	AVE	CR
Nostalgia as sport experience	NSE1	0.727	0.816	0.871	0.574
	NSE2	0.704			
	NSE3	0.795			
	NSE4	0.764			
	NSE5	0.796			
Nostalgia as environment	NE2	0.704	0.842	0.884	0.562
	NE3	0.629			
	NE4	0.796			
	NE5	0.835			
	NE6	0.695			
Nostalgia as socialization	NS1	0.888	0.92	0.938	0.719
	NS2	0.885			
	NS3	0.902			
	NS4	0.902			
	NS5	0.67			
	NS6	0.815			
Nostalgia as personal identity	NPI1	0.809	0.909	0.928	0.647
	NPI2	0.843			
	NPI3	0.831			
	NPI4	0.817			
	NPI5	0.814			
	NPI6	0.74			
	NPI7	0.77			
Nostalgia as group identity	NGI1	0.775	0.894	0.913	0.57
	NGI2	0.842			
	NGI3	0.726			
	NGI4	0.827			
	NGI5	0.777			
	NGI6	0.664			
	NGI7	0.674			
	NGI8	0.737			
Reconditioning	RC1	0.844	0.771	0.865	0.681
	RC2	0.826			
	RC3	0.807			
Stimulus control	SC1	0.909	0.885	0.928	0.811
	SC2	0.912			
	SC3	0.881			
Behavioural monitoring	BM1	0.947	0.922	0.951	0.866
	BM2	0.918			
	BM3	0.926			
Subjective well-being	SWB1	0.765	0.877	0.91	0.671
	SWB2	0.797			
	SWB3	0.822			
	SWB6	0.768			
	SWB7	0.861			

AVE = average variance extracted; CR = composite reliability.

**Table 2:** Assessment of measurement model: higher-order constructs.

Second-Order Construct	Indicators	Factor Loadings	Cronbach’s α	AVE	CR
Nostalgia for sport activities	NSE	0.663	0.828	0.88	0.597
	NE	0.881			
	NS	0.79			
	NPI	0.819			
	NGI	0.689			
Self-regulation	RC	0.887	0.873	0.922	0.797
	SC	0.9			
	BM	0.891			

AVE = average variance extracted; CR = composite reliability.

**Table 3:** Discriminant validity—HTMT.

Constructs	1	2	3	4	5	6	7	8	9
Nostalgia as sport experience									
Nostalgia as environment	0.505								
Nostalgia as socialization	0.539	0.285							
Nostalgia as personal identity	0.653	0.579	0.389						
Nostalgia as group identity	0.72	0.507	0.705	0.669					
Reconditioning	0.612	0.414	0.343	0.614	0.471				
Stimulus control	0.448	0.505	0.204	0.568	0.449	0.86			
Behavioural monitoring	0.463	0.343	0.317	0.515	0.608	0.79	0.796		
Subjective well-being	0.219	0.177	0.482	0.345	0.442	0.39	0.367	0.463	

**Table 4:** Results of the hypothesized model.

Path	*β*	SE	*t*-Value
H1: Nostalgia → Self-regulation	0.602	0.083	7.258 ***
H2: Nostalgia → Subjective well-being	0.239	0.117	2.049 *
H3: Self-regulation → Subjective well-being	0.256	0.105	2.424 *

Note. * *p* < 0.05; *** *p* < 0.001; SE = standard error.

## Data Availability

Data presented in this study are available upon request from the corresponding author. The data are not publicly available because of privacy concerns.
